# Hormonal Contraception, Menstrual Cycle Characteristics, and Lower Limb Injuries in Elite Female Team Sports—Identifying Factors Associated With Increased Injury Prevalence: A Cross‐Sectional Study

**DOI:** 10.1002/hsr2.71812

**Published:** 2026-02-15

**Authors:** Elisabeth Maria Kirschbaum, Jana Henke, Katrin Heyde, Kirsten Legerlotz

**Affiliations:** ^1^ Movement Biomechanics, Institute of Sport Science Humboldt‐Universität zu Berlin Berlin Germany; ^2^ Research Group Field Hockey, Institute for Applied Training Science Leipzig Germany; ^3^ Department of Biomechanics in Sports, Faculty of Sports Science University of Leipzig Leipzig Germany; ^4^ Department of Movement and Training Science, Institute of Sport Science University of Wuppertal Wuppertal Germany

**Keywords:** ACL, basketball, field hockey, football, handball, prevention, volleyball, women

## Abstract

**Background and Aims:**

This study aimed to examine the prevalence of menstrual cycle characteristics and lower limb injuries among elite female team sport athletes, and to identify factors influencing injury risk.

**Methods:**

Data were collected from 301 female team athletes across the German 1st and 2nd division in basketball, field hockey, football, handball, and volleyball using an online questionnaire. Information was gathered on gynaecological health and lower extremity injuries in the past 12 months.

**Results:**

Forty‐two percent used hormonal contraceptives, while among non‐users, 81% were naturally menstruating and 19% had a menstrual dysfunction (MD). Of those naturally menstruating, 46% experienced dysmenorrhea in every menstrual cycle, and 18% took painkillers during each menstruation. Regarding injuries, 42% reported at least one lower limb injury in the past 12 months, with 44% classified as severe. The most commonly affected regions were the knee (44%), ankle (31%), and upper thigh (9%). MD was associated with lower anterior cruciate ligament (ACL) injury rates (*p* = 0.04, Cramer's *V* = 0.199, 95% CI: −∞ to −0.09), while regular periodic health examinations (PHE) were associated with higher injury rates (*p* = 0.05, Cramer's *V* = 0.121, 95% CI: −0.98 to −0.01), particularly knee cartilage injuries (*p* = 0.001, Cramer's *V* = 0.241, 95% CI: −3.19 to −0.59). No significant associations were found between injury rates and dysmenorrhea or premenstrual syndrome.

**Conclusion:**

This study highlights the complex relationship between menstrual cycle characteristics and injury risk in female athletes, showing MD's unexpected association with lower ACL injury rates. Further, it emphasizes the need for targeted injury prevention programs, regular PHE, and enhanced medical support structures to reduce injury risks in elite female team sports.

## 1 Background

It is well known that anterior cruciate ligament (ACL) injuries occur more frequently in female than in male team sport athletes [1, 2, 3], raising concerns about sex‐specific risk factors. Both menstrual cycle (MC) phase and hormonal contraceptive (HC) use have been identified as factors influencing ACL injury risk [4, 5, 6]. However, the underlying mechanisms remain unclear, and causal relationships have yet to be established [7]. Moreover, research has primarily focused on ACL injuries, while comparatively little is known about other types of lower limb injuries.

Furthermore, findings on the relationship between the MC phase and other types of injuries are inconsistent. Some studies report higher rates of muscle and tendon injuries in the late follicular phase [8, 9] while others observed a higher incidence during the luteal phase [10, 11]. Beyond the phase itself, MC characteristics such as menstrual dysfunctions (MD) and prolonged MC length have been tied to injury risk. Extended cycles have been associated with higher injury frequency [9] and athletes experiencing MD are more prone to time‐loss injuries [12]. MD is a core symptom of relative energy deficiency in sport (REDs) complex, caused by low energy availability and associated with negative health and performance outcomes [13]. REDs contributes to an increased injury risk through impaired physiological and psychological function, resulting in higher rates of time‐loss injuries and stress fractures [13].

Additionally, many elite female athletes report experiencing discomfort and pain in the context of the MC, such as the premenstrual syndrome (PMS) or dysmenorrhea [14, 15], which may also affect injury risk. For example, females with PMS have exhibited impaired balance during the late luteal phase [16], suggesting that such symptoms may affect neuromuscular function [17] and potentially increase injury susceptibility. HC users have reported self‐perceived balance impairments during the inactive pill phase, and similar impairments have been observed in naturally menstruating individuals during the early follicular phase [18]. Thus, different hormonal concentrations related to the MC, dysmenorrhea or HC may affect injury risk in multiple ways.

In addition, gendered disparities in access to sport and training environments may further contribute to the increased injury risk observed in female athletes [19]. Female team sport athletes often face limited access to medical support structures [20, 21], with a lower medical staff‐to‐player ratio in professional women's football compared to men, potentially affecting injury prevention, treatment, and rehabilitation procedures [22]. Regular Periodic Health Examinations (PHE) during pre‐season and in‐season periods is considered essential for assessing and managing injury risk [23], and may differ between male and female elite sports.

The aim of this study was (1) to describe the prevalence of different hormonal profiles and dysmenorrhea, and lower limb injuries among elite female team sport athletes, and (2) to identify factors associated with lower limb injuries. We hypothesized that MD, dysmenorrhea, and PMS would be associated with higher injury rates, while annual PHE, such as injury risk factor assessments and sports medical check‐ups, would be associated with lower injury rates.

## 2 Methods

### 2.1 Study Design, Participants, and Recruitment Strategy

**Figure 1:**
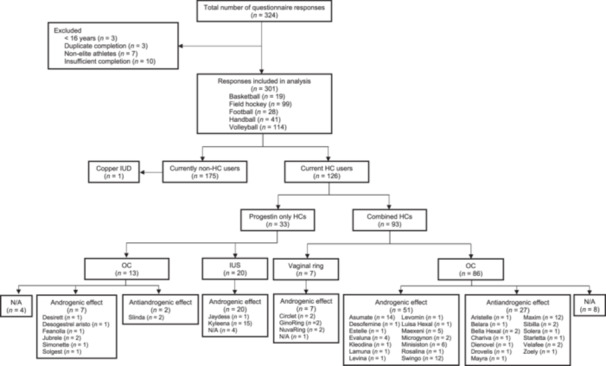
Overview of the athletes included in the study, frequency of non‐HC users and type, delivery method, effect of progestins, and frequency of HC user. HC, hormonal contraceptives; IUD, intrauterine device; IUS, intrauterine system; N/A, not applicable; OC, oral contraceptives.

This cross‐sectional study analysed self‐reported gynaecological health characteristics and lower limb injuries among elite female team sport athletes. Female athletes were eligible to participate if they were currently competing in the German 1st or 2nd division in basketball, football, field hockey, handball or volleyball and were 16 years or older. Participants were recruited through national leagues, email correspondence with clubs, and social media. Recruitment and data collection were from February 2023 until April 2023. Initially, 324 athletes completed the questionnaire. After exclusion of faulty data sets (Figure 1), a total of 301 elite (tier 4, *n* = 93) and highly trained (tier 3, *n* = 208) [24] female team sport athletes were included in the final analysis. The study was reviewed and approved by the ethical review board of the Institute for Applied Training Science (ER_2023.06.01_4). Written informed consent was obtained from all participants. This study adheres to the Strengthening the Reporting of Observational Studies in Epidemiology (STROBE) guidelines (Supporting Information S1) [25].

### 2.2 Questionnaire

The online questionnaire (SoSci Survey GmbH, Munich, Germany) gathered data in German language about demographics, lower extremity injuries in the past 12 months, and gynaecological health (Supporting Information S2 and S3).

The questions on demographics included general personal data, and sports‐related information about sports discipline, professional status, weekly training volume, experience at the elite and competition level, annual injury risk factor assessment, and annual sports medical check‐ups.

To record lower limb injuries in the past 12 months, a drop‐down list was applied according to the International Olympic Committee (IOC) guidelines [26]. The athletes reported the affected body area, the injured tissue, the pathology type, and the time loss. An open‐ended option was also provided for pathology type, allowing athletes to specify their injury. These responses were then categorized by EMK into predefined categories

The gynaecological health data part contained the German version of the International Consultation on Incontinence Questionnaire‐Urinary Incontinence Short Form (ICIQ‐UI SF) [27] and a questionnaire on gynaecological health characteristics which has been used in a previous study [15].

The questionnaire was pretested by a group of female athletes (*n* = 6), sport scientists (*n* = 2), and a medical doctor (*n* = 1).

### 2.3 Data Analysis

Injury characteristics and severity were classified according to the IOC guidelines [26] and categorized by body area, tissue type, and injury type. Severity was determined based on the time loss reported by athletes and classified as slight (0 days), mild (1–7 days), moderate (8–28 days), or severe (> 28 days) [26].

Gynaecological age was calculated as the current age minus the age at menarche [28]. Non‐HC users were classified as naturally menstruating if their MC length ranged between 25 and 35 days [29]; otherwise, they were categorized as having MD, including primary amenorrhea (absence of menarche), oligomenorrhea (MC length > 35 and ≤ 90 days), secondary amenorrhea (MC length > 90 days), and polymenorrhea (MC length < 25 days) [30].

### 2.4 Statistical Analysis

All analyses were conducted using MS Excel (Microsoft Corp, Redmond, WA, USA), IBM SPSS Statistics for Windows, version 29 (IBM Corp., Armonk, NY, USA) and JASP, version 0.95.4 (JASP team, Amsterdam, The Netherlands). The Shapiro–Wilk test was used to test for normality.

To analyse differences between sports in continuous variables (e.g., demographics), one‐way ANOVA with Bonferroni post‐hoc correction was used for normally distributed data, with partial eta‐squared (ηp²) reported as the effect size. For non‐normally distributed variables, the Kruskal–Wallis test was applied, with effect sizes reported as *r*. Categorical data (e.g., frequency of PHE or injuries) were compared using *χ* ^2^ tests with Fisher's exact test and Bonferroni‐adjusted post‐hoc tests, with effect sizes reported as Cramer's *V*.

Group comparisons between, for example, injured and non‐injured players were performed using independent‐samples *t*‐tests for normally distributed variables, and Mann–Whitney *U* tests for non‐normally distributed variables, with effect sizes *r* reported. Pairwise comparisons of categorical variables were conducted using *χ* ^2^ tests with Fisher's exact test and Bonferroni correction, with effect size reported as Cramer's *V*.

Continuous data were presented as mean (SD) if normally distributed and as median (IQR) if not normally distributed. Categorical data were reported as frequencies or prevalence.

An a priori significance level of *α* ≤ 0.05 was applied. All analyses were two‐sided. Results are presented as *p* values with associated effect sizes and 95% confidence intervals (CI). Effect sizes' interpretation thresholds followed established guidelines: for ηp² = 0.01 (small), 0.06 (medium), and 0.14 (large); for Cramer's *V* = 0.10 (small), 0.30 (medium), 0.50 (large) [31]. For *r*, we applied current recommendations in injury prevention research: *r* = 0.10 (small), 0.40 (medium), 0.80 (large) [32].

All analyses and reporting were conducted in accordance with the Statistical Analyses and Methods in the Published Literature (SAMPL) guidelines [33] and recommendations for clinical research reporting [34].

## 3 Results

### 3.1 Participants Characteristics

Demographics and sports‐related information of 301 elite female team sport athletes are presented in Table 1, along with significant differences across sports in the proportion of professional players (*p* < 0.001, Cramer's *V* = 0.280), weekly training volume (*p* = 0.006, *r* = 0.035), weekly prevention training volume (*p* = 0.02, *r* = 0.025), frequency of annual injury risk factor assessments (*p* < 0.001, Cramer's *V* = 0.406) and annual sports medical check‐ups (*p* < 0.001, Cramer's *V* = 0.556).

**Table 1:** Demographic data from participants classified into sports disciplines.

	Total *n* = 301	Basketball *n* = 19	Field hockey *n* = 99	Football *n* = 28	Handball *n* = 41	Volleyball *n* = 114	*p* value	Effect size^f^
Age, years	22.0 (19.0–25.5)	23.0 (18.0–27.0)	22.0 (18.0–25.0)	22.0 (20.0–25.8)	22.0 (21.0–24.0)	23.0 (19.0–27.0)	0.12	0.011
Height, cm	174.1 (7.8)	176.4 (7.7)^b^, * ^,^ c (*p* = 0.001)	169.4 (5.8)^a^, * ^,^ d, * ^,^ e, *	168.8 (6.9)^a^ (*p* = 0.001),^d^ (*p* = 0.006),^e^, *	174.3 (5.5)^b^, * ^,^ c (*p* = 0.006),^e^ (*p *= 0.001)	178.9 (7.2)^b^, * ^,^ c, * ^,^ d (*p* = 0.001)	< 0.001	0.323^g^
Weight, kg	67.0 (62.0–72.5)	66.0 (63.0‐75.0)	63.0 (59.0–69.0)^d^, * ^,^ e, *	63.0 (60.0–70.0)^d^ (*p* = 0.001),^e^ (*p *= 0.01)	72.0 (67.5–78.0)^b^, * ^,^ c (*p* = 0.001)	70.0 (65.0–75.0)^b^, * ^,^ c (*p *= 0.014)	< 0.001	0.174
BMI, kg/m²	22.3 (2.0)	21.9 (2.2)^d^ (*p *= 0.004)	22.1 (2.0)^d^, *	22.8 (1.4)	23.8 (1.9)^a^ (*p *= 0.004),^b^, * ^,^ e, *	21.9 (1.9)^d^, *	< 0.001	0.102^g^
Professional players	18 (6%)	1 (5%)	1 (1%)^c^, *	7 (25%)^b^, * ^,^ d (*p *= 0.006)	1 (2%)^c^ (*p* = 0.006)	8 (7%)	< 0.001	0.280^h^
Experience at the elite level, years	9.0 (6.9–13.0)	9.0 (6.0–11.0)	10.0 (6.0–15.0)	10.5 (7.0–15.0)	10.0 (5.5–12.5)	8.0 (5.0–12.0)	0.13	0.011
Experience at the competition level, years	3.0 (1.5–6.0)	3.0 (2.0–6.0)	4.0 (1.0–7.0)	4.0 (2.3–8.5)	3.0 (1.8–5.0)	3.0 (1.9–6.0)	0.50	0.002
Self‐reported total weekly training volume, h	13.0 (11.0–17.0)	13.0 (11.0–17.0)	12.0 (10.0–15.0)^e^ (*p* = 0.02)	13.0 (12.0–16.8)	14.0 (11.0–17.0)	13.3 (11.0–18.0)^b^ (*p* = 0.015)	0.006	0.035
Self‐reported total weekly prevention training volume, h	1.0 (0.4–1.8)	0.6 (0.0–1.0)^c^ (*p *= 0.02)	1.0 (0.5–1.6)	1.5 (0.8–3.1)^a^ (*p* = 0.02)	1.0 (0.5–1.5)	1.0 (0.4–2.0)	0.02	0.025
Annual injury risk factor assessment	119 (40%)	4 (21%)^d^, *	34 (34%)^d^, *	17 (61%)^e^, *	34 (83%)^a^, * ^,^ b, * ^,^ e, *	30 (26%)^d^, * ^,^ e, *	< 0.001	0.406^h^
Annual sports medical check‐up	147 (49%)	13 (68%)^b^ (*p *= 0.004),^c^ (*p* = 0.003),^d^ (*p *= 0.01),^e^ (*p* = 0.004)	31 (31%) (*p *= 0.004),^c^, * ^,^ d, *	28 (100%)^a^ (*p* = 0.003),^b^, * ^,^ e, *	39 (95%)^a^ (*p *= 0.010),^b^, * ^,^ e, *	36 (32%)^a^ (*p* = 0.004),^c^, * ^,^ d, *	< 0.001	0.556^h^

*Note:* Continuous data is presented in the form mean (SD) or median (IQR), and categorical data is presented as *n* (%).

Abbreviations: BMI, body mass index; CI, confidence interval; *n* (%), sample size (percentage of).

^a^ different from basketball.

^b^ different from field hockey.

^c^ different from football.

^d^ different from handball.

^e^ different from volleyball.

^f^ indicates effect size *r.*

^g^ indicates effect size ηp².

^h^ indicates effect size Cramer's *V*.

* indicates a significant difference with *p* < 0.001.

### 3.2 Overall Gynaecological Health Characteristics

**Figure 2:**
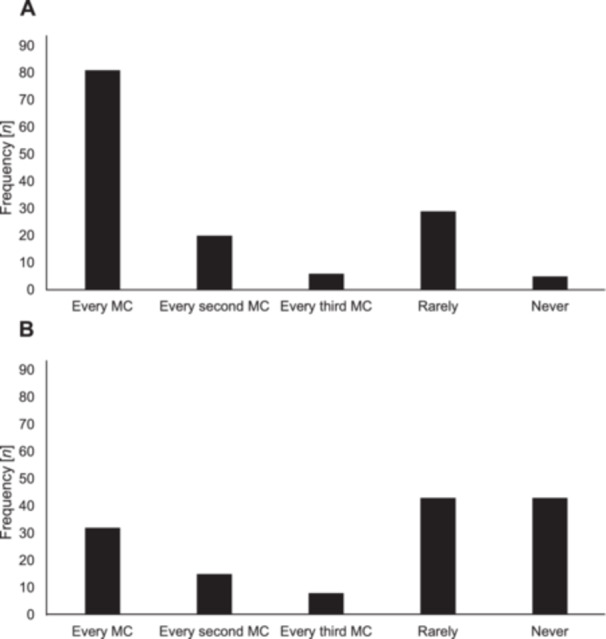
Overview of the frequency of (A) dysmenorrhea and (B) painkiller intake during menstruation among naturally menstruating athletes (*n* = 175). MC, menstrual cycle.

The median age at menarche was 13.0 years (IQR 12.0–14.0), and the median gynaecological age was 9.0 years (IQR 6.0–12.0), with no significant differences between sports in age at menarche (*p* = 0.31, *r* = 0.003) and gynaecological age (*p* = 0.08, *r* = 0.015). Forty‐two percent (*n* = 126) of the athletes used HC, while 58% (*n *= 175) were non‐HC users (Supporting Information S4). Among HC users (*n* = 126), 68% used combined oral contraceptives (*n* = 86), 16% intrauterine systems (*n* = 20), 10% progestin‐only oral contraceptives (*n* = 13), and 6% a vaginal ring (*n* = 7). Among non‐HC users, 81% were naturally menstruating (*n* = 141), while 9% had polymenorrhea (*n* = 15), 6% oligomenorrhea (*n* = 11), 2% primary amenorrhea (*n* = 4), and 2% secondary amenorrhea (*n* = 4). The median weekly training volume did not differ significantly between those who were naturally menstruating (8.0 h, IQR 7.0–11.0) and those with any form of MD (8.0 h, IQR 6.3–12.8; *p* = 0.56, *r* = 0.045). Among naturally menstruating athletes (*n* = 141), 48% (*n* = 85) reported experiencing PMS with no significant differences between sports (*p* = 0.10, Cramer's *V* = 0.238). Additionally, 46% (*n* = 81) reported experiencing dysmenorrhea during every MC, while 3% (*n* = 5) never experienced dysmenorrhea. Furthermore, 18% (*n* = 32) of the naturally menstruating athletes reported taking painkillers during every menstruation, whereas 50% (*n *= 86) rarely or never used painkillers (Figure 2).

Moreover, 75% (*n* = 225) of all athletes had a gynaecological check‐up at least once a year. The median ICIQ‐UI SF score was 0.0 (IQR 0.0–3.0) and the prevalence of stress urinary incontinence was 27% (*n* = 81). There were no significant differences between sports in the frequency of gynaecological check‐ups (*p* = 0.13, Cramer's *V* = 0.154), ICIQ‐UI SF scores (*p* = 0.57, *r* = 0.004), or prevalence of stress urinary incontinence (*p* = 0.57, Cramer's *V* = 0.097).

### 3.3 Injury Characteristics

Of the 301 athletes, 42% (*n* = 127) reported at least one lower limb injury in the past 12 months. A total of 180 injuries were reported. No significant differences across sports were detected for the frequency of injured athletes (*p* = 0.10, Cramer's *V* = 0.161), the distribution of time‐loss and non‐time‐loss injuries (*p* = 0.73, Cramer's *V* = 0.108) or in injuries affecting the dominant versus non‐dominant leg (*p* = 0.70, Cramer's *V* = 0.111). The most frequently injured body area was the knee (44%, *n* = 79), followed by the ankle (31%, *n* = 31) and upper thigh (9%, *n *= 16) (Table 2).

**Table 2:** Incidence, total time loss, and cumulative severity score of injuries.

	Total	Basketball	Field hockey	Football	Handball	Volleyball
Total athletes, *n*	301	19	99	28	41	144
Total injured athletes, *n* (%)	127 (42%)	12 (63%)	42 (42%)	14 (50%)	20 (49%)	40 (35%)
Total injuries, *n*	180	21	52	17	31	59
Total time loss injuries, *n* (%)	154 (86%)	18 (86%)	45 (87%)	15 (88%)	24 (77%)	52 (88%)
Total non‐time loss injuries, *n* (%)	26 (14%)	3 (14%)	7 (14%)	2 (12%)	7 (23%)	7 (12%)
Dominant leg, *n* (%)	86 (48%)	11 (52%)	26 (50%)	10 (59%)	12 (39%)	27 (46%)
Non‐dominant leg, *n* (%)	94 (52%)	10 (48%)	26 (50%)	7 (41%)	19 (61%)	32 (54%)
Most common body area injured						
Hip/groin, *n* (%)	11 (6%)	1 (5%)	5 (10%)	1 (6%)	2 (7%)	2 (3%)
Upper thigh, *n* (%)	16 (9%)	2 (10%)	7 (14%)	1 (6%)	2 (7%)	4 (7%)
Knee, *n* (%)	79 (44%)	8 (38%)	21 (40%)	9 (53%)	10 (32%)	31 (53%)
Lower thigh, *n* (%)	10 (6%)	1 (5%)	2 (4%)	1 (6%)	3 (10%)	3 (5%)
Ankle, *n* (%)	55 (31%)	6 (29%)	16 (31%)	4 (24%)	11 (36%)	18 (31%)
Foot, *n* (%)	9 (5%)	3 (14%)	1 (2%)	1 (6%)	3 (10%)	1 (2%)

*Note: n* (%), sample size (percentage of). Data is presented in the form *n* (%).

Most injuries (44%) were classified as severe, 23% as moderate, 18% as mild, and 14% as non‐time loss injuries (Table 3).

**Table 3:** Severity of time loss of injuries by region, tissue, and pathology type.

	Cases (*n*)	Slight (0 days)	Mild (1–7 days)	Moderate (8–28 days)	Severe (> 28 days)
Total Injuries	180 (100%)	26 (14%)	33 (18%)	41 (23%)	80 (44%)
Hip/Groin, *n* (%)	11 (6%)	3 (2%)	5 (3%)	1 (1%)	2 (1%)
Muscle/tendon					
Muscle injury, *n* (%)	6 (3%)	3 (2%)	2 (1%)	1 (1%)	
Nervous					
Peripheral nerve injury, *n* (%)	1 (1%)		1 (1%)		
Cartilage/synovium/bursa					
Cartilage injury, *n* (%)	1 (1%)				1 (1%)
Nonspecific, *n* (%)	3 (2%)		2 (1%)		1 (1%)
Upper thigh, *n* (%)	16 (9%)	2 (1%)	6 (3%)	6 (3%)	2 (1%)
Muscle/tendon					
Muscle injury, *n* (%)	14 (8%)	2 (1%)	6 (3%)	5 (3%)	1 (1%)
Tendinopathy, *n* (%)	1 (1%)				1 (1%)
Tendon rupture, *n* (%)	1 (1%)			1 (1%)	
Knee, *n* (%)	79 (44%)	10 (6%)	9 (5%)	9 (5%)	51 (28%)
Muscle/tendon					
Muscle injury, *n* (%)	2 (1%)		1 (1%)	1 (1%)	
Tendinopathy, *n* (%)	10 (6%)	2 (1%)	2 (1%)	3 (2%)	3 (2%)
Bone					
Bone contusion, *n* (%)	1 (1%)				1 (1%)
Cartilage/synovium/bursa					
Cartilage injury, *n* (%)	25 (14%)	4 (2%)		2 (1%)	19 (11%)
Synovitis/capsulitis, *n* (%)	3 (2%)		2 (1%)		1 (1%)
Bursitis, *n* (%)	6 (3%)	2 (1%)	2 (1%)	1 (1%)	1 (1%)
Ligament/joint capsule					
Joint sprain, *n* (%)	8 (4%)	1 (1%)	1 (1%)	1 (1%)	5 (3%)
ACL injury, *n* (%)	23 (13%)		1 (1%)	1 (1%)	21 (12%)
Chronic instability, *n* (%)	1 (1%)	1 (1%)			
Lower thigh, *n* (%)	10 (6%)	1 (1%)	1 (1%)	5 (3%)	3 (2%)
Muscle/tendon					
Muscle injury, *n* (%)	3 (2%)			3 (2%)	
Tendinopathy, *n* (%)	1 (1%)				1 (1%)
Bone					
Bone stress injury, *n* (%)	5 (3%)		1 (1%)	2 (1%)	2 (1%)
Nonspecific, *n* (%)	1 (1%)	1 (1%)			
Ankle, *n* (%)	55 (31%)	7 (4%)	11 (6%)	19 (11%)	18 (10%)
Muscle/tendon					
Muscle injury, *n* (%)	1 (1%)	1 (1%)			
Muscle contusion, *n* (%)	1 (1%)		1 (1%)		
Tendinopathy, *n* (%)	1 (1%)		1 (1%)		
Tendon rupture, *n* (%)	2 (1%)		1 (1%)	1 (1%)	
Bone					
Bone stress injury, *n* (%)	1 (1%)				1 (1%)
Cartilage/synovium/bursa					
Cartilage injury, *n* (%)	1 (1%)				1 (1%)
Synovitis/capsulitis, *n* (%)	1 (1%)			1 (1%)	
Bursitis, *n* (%)	1 (1%)				1 (1%)
Ligament/joint capsule					
Joint sprain (ligament tear), *n* (%)	36 (20%)	4 (2%)	2 (1%)	15 (8%)	15 (8%)
Joint sprain (acute instability episode), *n* (%)	8 (4%)	1 (1%)	5 (3%)	2 (1%)	
Chronic instability, *n* (%)	2 (1%)	1 (1%)	1 (1%)		
Foot, *n* (%)	9 (5%)	3 (2%)	1 (1%)	1 (1%)	4 (2%)
Muscle/tendon					
Muscle injury, *n* (%)	2 (1%)	1 (1%)	1 (1%)		
Muscle contusion, *n* (%)	1 (1%)	1 (1%)			
Tendinopathy, *n* (%)	2 (1%)	1 (1%)		1 (1%)	
Bone					
Fracture, *n* (%)	1 (1%)				1 (1%)
Bone stress injury, *n* (%)	2 (1%)				2 (1%)
Cartilage/synovium/bursa					
Bursitis, *n* (%)	1 (1%)				1 (1%)

The three most frequent injuries were ankle joint sprains (*n* = 36), knee cartilage injuries (*n* = 25) and ACL injuries (*n* = 23). Two athletes reported experiencing two separate ankle sprains, while three reported sustaining two knee cartilage injuries.

### 3.4 Factors Associated With Injuries

Compared to non‐injured athletes (*n* = 174), injured athletes (*n* = 127) reported significantly higher weekly training volumes (*p* = 0.03, *r* = 0.124, 95% CI: 0.00 to 0.26) and more annual sports medical check‐ups (*p* = 0.05, Cramer's *V* = 0.121, 95% CI: −0.98 to −0.01). Athletes with ACL injuries (*n* = 23) reported significantly higher weekly training volumes (*p* = 0.02, *r* = 0.160, 95% CI: 0.08 to 0.54) and significantly lower prevalence of MD (*p* = 0.04, Cramer's *V* = 0.199, 95% CI: −∞ to −0.09) compared to non‐injured athletes. Athletes with knee cartilage injuries (*n* = 22) reported significantly higher BMIs (*p* = 0.008, *r *= 0.190, 95% CI: −2.08 to −0.32) and had annual sports medical check‐ups more frequently (*p* = 0.001, Cramer's *V* = 0.241, 95% CI: −3.19 to −0.59) compared to non‐injured athletes. Detailed information are presented in Tables 4 and 5.

**Table 4:** Participant and gynaecological health characteristics divided into injured and non‐injured players.

	Non‐injured athletes vs.	Injured athletes	*p* value	Effect size^f^	95% CI
***n***	174	127			
Age, years	22.0 (19.0–26.0)	22.0 (19.0–25.0)	0.30	0.060	−0.20 to 0.06
Gynaecological age^a^, years	9.0 (6.0–12.0)	9.0 (5.5–12.0)	0.21	0.073	−0.22 to 0.05
Age at menarche^a^, years	13.0 (12.0–14.0)	13.0 (12.0–14.0)	0.86	0.006	−0.12 to 0.14
Height, cm	174.2 (7.7)	174.0 (8.0)	0.81	0.014	−1.58 to 2.02
Weight, kg	67.2 (8.4)	68.3 (7.8)	0.25	0.067	−2.97 to 0.76
BMI, kg/m²	22.1 (2.0)	22.6 (2.1)	0.05	0.113	−0.92 to 0.00
Weekly training volume, h	13.0 (11.0–16.0)	13.5 (12.0–18.0)	0.03	0.124	0.00 to 0.26
Weekly prevention training volume, h	0.9 (0.3–1.7)	1.0 (0.5–2.0)	0.06	0.107	−0.01 to 0.26
Experience in elite sports, years	9.0 (5.0–13.0)	9.0 (6.0–13.0)	> 0.99	0.000	−0.13 to 0.13
Experience at competition level, years	3.0 (1.9–6.0)	3.0 (1.0–6.0)	0.89	0.008	−0.14 to 0.12
Professional athletes, *n* (%)	11 (6%)	7 (6%)	0.81	0.017^g^	−1.29 to 0.93
National players, *n* (%)	17 (10%)	20 (16%)	0.16	0.090^g^	−0.20 to 1.30
Annual injury risk factor assessment, *n* (%)	64 (37%)	55 (43%)	0.28	0.066^g^	−0.77 to 0.22
Annual sports medical check‐up, *n* (%)	76 (44%)	71 (56%)	0.05	0.121^g^	−0.98 to −0.01
SUI, *n* (%)	45 (26%)	36 (28%)	0.69	0.028^g^	−0.42 to 0.67
ICIQ‐UI SF, score	0.0 (0.0–3.0)	0.0 (0.0–3.0)	0.99	0.001	−0.13 to 0.13
Menstrual dysfunction^b^, *n* (%)	25 (24%)	9 (13%)	0.08	0.136^g^	−1.71 to 0.13
HC, *n* (%)	69 (40%)	57 (45%)	0.41	0.052^g^	−0.28 to 0.70
Type of HC^c^, ^d^			0.69	0.039^g^	−0.79 to 1.05
Progestin‐only HC^c^, ^d^, *n* (%)	17 (25%)	16 (28%)			
Combined HC^c^, ^d^, *n* (%)	52 (75%)	41 (72%)			
Effect of synthetic progestins^c^, ^d^ ^,^ ^c^, ^d^			0.39	0.100^g^	−0.47 to 1.44
Antiandrogenic^c^, ^d^ ^,^ ^c^, ^d^, *n* (%)	43 (71%)	42 (80%)			
Androgenic^c^, ^d^ ^,^ ^c^, ^d^, *n* (%)	18 (30%)	11 (21%)			
PMS^b^ ^,^ ^e^, *n* (%)	34 (43%)	34 (56%)	0.13	0.131^g^	−1.26 to 0.19

*Note:* Continuous data is presented in the form mean (SD) or median (IQR), and categorical data is presented as *n* (%).

Abbreviations: BMI, body mass index; CI, confidence interval; HC, hormonal contraceptives; ICIQ‐UI SF, incontinence questionnaire‐urinary incontinence short form; n (%), sample size (percentage of); PMS, premenstrual syndrome; SUI, stress urinary incontinence.

^a^ Participants who did not had their menarche were excluded from the analysis.

^b^ Participants who are currently HC users were excluded.

^c^ Participants who are currently non‐HC users were excluded.

^d^ Participants who did not report their brand were excluded from the analysis.

^e^ Participants who are currently not naturally menstruating were excluded.

^f^ indicates effect size *r*.

^g^ indicates effect size Cramer's *V*.

**Table 5:** Participant and gynaecological health characteristics divided into non‐injured players (*n* = 174) and players reporting the three most common injury types: ankle joint sprain (*n* = 34), ACL injury (*n* = 23) and knee cartilage injury (*n* = 22).

	Non‐injured athletes vs.	Ankle joint sprain				ACL injury				Knee cartilage injury			
			*p* value	Effect size^f^	95% CI		*p* value	Effect size^f^	95% CI		*p* value	Effect size^f^	95% CI
***n***	174	34				23				22			
Age, year	22.0 (19.0–26.0)	20.5 (19.0–25.0)	0.21	0.087	−0.34 to 0.08	21.0 (19.0–24.0)	0.21	0.090	−0.39 to 0.09	21.5 (19.8–25.5)	0.93	0.006	−0.26 to 0.24
Gynaecological age^a^, year	9.0 (6.0–12.0)	7.5 (5.0–12.0)	0.19	0.030	−0.34 to 0.07	7.0 (4.0–11.0)	0.09	0.123	−0.44 to 0.03	9.0 (5.5–13.5)	0.89	0.074	−0.24 to 0.27
Age at menarche^a^, year	13.0 (12.0–14.0)	13.0 (13.0–14.0)	0.66	0.091	−0.17 to 0.25	14.0 (12.5–15.0)	0.24	0.084	−0.10 to 0.38	13.0 (12.0–14.0)	0.31	0.010	−0.38 to 0.13
Height, cm	174.2 (7.7)	175.1 (8.0)	0.54	0.043	−3.79 to 1.97	175.5 (7.8)	0.44	0.055	−4.72 to 2.06	172.6 (9.4)	0.46	0.148	−1.98 to 5.11
Weight, kg	67.2 (8.4)	69.2 (7.3)	0.20	0.090	−5.07 to 0.99	68.0 (63.0–70.0)	0.78	0.021	−0.21 to 0.28	68.0 (63.0–73.0)	0.38	0.064	−0.14 to 0.36
BMI, kg/m²	22.1 (2.0)	22.6 (1.8)	0.19	0.092	−1.19 to 0.25	22.2 (2.2)	0.87	0.012	−0.95 to 0.80	23.3 (2.0)	0.008	0.190	−2.08 to −0.32
Weekly training volume, h	13.0 (11.0–16.0)	13.5 (11.4–18.5)	0.10	0.114	−0.11 to 0.31	16.0 (12.0–18.0)	0.02	0.160	0.08 to 0.54	14.0 (12.8–18.0)	0.09	0.120	−0.07 to 0.42
Weekly prevention training volume, h	0.9 (0.3–1.7)	1.0 (0.3–2.0)	0.39	0.059	−0.12 to 0.30	1.0 (0.5–2.0)	0.34	0.068	−0.13 to 0.36	1.1 (0.7–2.6)	0.05	0.138	0.00 to 0.47
Experience in elite sports, year	9.0 (5.0–13.0)	7.0 (5.0–10.0)	0.11	0.110	−0.37 to 0.04	10.0 (6.0–13.0)	0.70	0.028	−0.20 to 0.29	11.0 (7.8–14.3)	0.19	0.094	−0.08 to 0.41
Experience at competition level, year	3.0 (1.9–6.0)	3.0 (1.4–5.0)	0.76	0.025	−0.25 to 0.17	4.0 (1.0–6.0)	0.86	0.013	−0.27 to 0.23	3.0 (1.0–5.3)	0.58	0.040	−0.32 to 0.18
Professional athletes, *n* (%)	11 (6%)	0 (0%)	0.22	0.104^g^	−∞ to 0.70	3 (13%)	0.21	0.084^g^	−1.01 to 2.24	3 (14%)	0.20	0.090^g^	−0.96 to 2.30
National players, *n* (%)	17 (10%)	3 (9%)	> 0.99	0.864^g^	−1.84 to 1.22	5 (22%)	0.15	0.122^g^	−0.42 to 2.13	4 (18%)	0.27	0.086^g^	−0.89 to 1.98
Annual injury risk factor assessment, *n* (%)	64 (37%)	14 (44%)	0.44	0.056^g^	−1.11 to 0.52	11 (48%)	0.36	0.073^g^	−1.42 to 0.53	11 (50%)	0.25	0.086^g^	−1.53 to 0.46
Annual sports medical check‐up, *n* (%)	76 (44%)	19 (56%)	0.26	0.091^g^	−1.31 to 0.31	13 (57%)	0.27	0.083^g^	−1.51 to 0.45	18 (82%)	0.001	0.241^g^	−3.19 to −0.59
SUI, *n* (%)	45 (26%)	13 (38%)	0.15	0.102^g^	−0.29 to 1.40	8 (35%)	0.45	0.065^g^	−0.65 to 1.42	4 (18%)	0.60	0.056^g^	−1.90 to 0.73
ICIQ‐UI SF, score	0.0 (0.0–3.0)	0.0 (0.0–3.0)	0.34	0.067	−0.13 to 0.29	0.0 (0.0–3.0)	0.59	0.035	−0.19 to 0.30	0.0 (0.0–0.0)	0.35	0.065	−0.33 to 0.16
Menstrual dysfunction^b^, *n* (%)	25 (24%)	2 (10%)	0.24	0.123^g^	−3.28 to 0.51	0 (0%)	0.04	0.199^g^	−∞ to −0.09	2 (15%)	0.73	0.063^g^	−2.83 to 1.08
HC, *n* (%)	69 (40%)	14 (41%)	> 0.99	0.011^g^	−0.77 to 0.87	7 (30%)	0.50	0.061^g^	−1.51 to 0.60	9 (41%)	> 0.99	0.008^g^	−0.98 to 1.04
Type of HC^c^			0.51	0.092^g^	−2.98 to 0.99		> 0.99	0.026^g^	−2.24 to 2.12		0.11	0.220^g^	−0.33 to 3.05
Progestin‐only HC^c^, *n* (%)	17 (25%)	2 (14%)				2 (29%)				5 (56%)			
Combined HC^c^, *n* (%)	52 (75%)	12 (86%)				5 (71%)				4 (44%)			
Effect of synthetic progestins^c^ ^,^ ^d^			0.53	0.074^g^	−1.79 to 1.10		0.66	0.103^g^	−1.31 to 4.80		10	0.226^g^	−0.32 to ∞
Antiandrogenic^c^ ^,^ ^d^, *n* (%)	43 (71%)	8 (62%)				6 (86%)				9 (100%)			
Androgenic^c^ ^,^ ^d^, *n* (%)	18 (30%)	5 (39%)				1 (14%)				0 (0%)			
PMS^b^ ^,^ ^e^, *n* (%)	34 (43%)	10 (56%)	0.43	0.102^g^	−1.70 to 0.63	4 (44%)	> 0.99	0.009^g^	−1.26 to 1.20	8 (73%)	10	0.198^g^	−3.11 to 0.25

*Note:* Continuous data is presented in the form mean (SD) or median (IQR), and categorical data is presented as *n* (%).

Abbreviations: BMI, body mass index; CI, confidence interval; HC, hormonal contraceptives; ICIQ‐UI SF, incontinence questionnaire‐urinary incontinence short form; *n* (%), sample size (percentage of); PMS, premenstrual syndrome; SUI, stress urinary incontinence.

^a^ Participants who did not had their menarche were excluded from the analysis.

^b^ Participants who are currently HC users were excluded.

^c^ Participants who are currently non‐HC users were excluded.

^d^ Participants who did not report their brand were excluded from the analysis.

^e^ Participants who are currently not naturally menstruating were excluded.

^f^ indicates effect size *r.*

^g^ indicates effect size Cramer's *V*.

## 4 Discussion

Our study revealed that 46% of the naturally menstruating elite female team sport athletes experienced dysmenorrhea in every MC and among non‐HC users 19% reported suffering from a MD. Contrary to our hypotheses, MD was associated with lower ACL injury rates, whereas annual PHE was associated with higher injury rates, especially knee cartilage injuries. No significant associations were found between injury rates and dysmenorrhea or PMS. Although most observed effects were small, they should not be dismissed in the context of elite sport [35, 36]. In elite female team sports, even marginal differences in injury incidence or severity can have meaningful implications for player availability and competitive performance, particularly in squads with limited depth [22]. While these effects may be trivial for the general population, they are interpreted in the context of elite sport in the subsequent discussion.

### 4.1 Overall Injuries

The findings of our study suggest that injury incidence and severity in elite female team sport athletes differs from males. We observed an overall injury incidence of 42%, with 14% resulting in no time loss and 44% being classified as severe. In contrast, data from elite male team sports in Germany showed a higher overall injury incidence of 73%, yet a lower proportion of severe injuries (14%) and a higher proportion of non–time‐loss injuries (47%) [37]. In contrast, a recent meta‐analysis [2] found no statistically significant sex‐based differences in injury severity, indicating that additional contextual factors may contribute to these disparities in our results.

One such factor may be the underreporting of slight or mild injuries in female athletes, particularly when self‐reported data are collected retrospectively. Previous research has shown that athletes may not perceive minor injuries as significant enough to report, especially when they do not result in time loss or require medical attention [38, 39]. Consequently, these injuries often remain undocumented, especially in settings where access to medical staff is limited [40]. Reliable injury surveillance depends heavily on the presence of experienced medical professionals [40]. Yet such resources are frequently lacking in elite female team sports environments [41]. This lack of medical infrastructure may also help explain the high proportion of severe injuries and extended absence periods in our cohort. In our sample, 92% of athletes were non‐professionals, which likely limited their ability to commit to comprehensive rehabilitation programs [20]. Furthermore, limited access to qualified medical staff, physiotherapists, and strength and conditioning professionals may have negatively impacted both immediate injury management and long‐term return‐to‐play outcomes [20, 21, 22, 41]. These findings underscore the importance of establishing professional support structures for elite female team sport athletes, not only to enhance return‐to‐play processes but also as a critical component of effective injury prevention strategies.

### 4.2 ACL Injuries and MD

Interestingly, our findings suggest MD to be associated with lower ACL injury rates. This outcome appears counterintuitive, given existing literature linking REDs with increased injury risk [13]. The nature of the injuries involved may in part explain this discrepancy. REDs is most commonly associated with overuse injuries such as stress fractures, which develop gradually under conditions of chronic energy deficiency and impaired bone health [13]. In contrast, ACL injuries typically result from acute and traumatic events during cutting, pivoting, or sudden deceleration movements [42].

In addition, ACL injuries are recognized as multifactorial in nature, with behavioural components such as motivation and risk‐taking playing an important role [7, 19]. Naturally menstruating athletes may experience more pronounced hormonal fluctuations compared to those with MD [43], which could influence both physiology and behaviour [7]. For instance, peaks in testosterone during ovulation have been associated with heightened risk‐taking tendencies [44]. Aggressive behaviour has been linked to a greater risk of injury in combat sports settings [45]. These findings suggest that cyclical hormonal variations in naturally menstruating athletes may contribute to injury risk, particularly for acute injuries like ACL tears, through behavioural factors.

Another possible explanation concerns the temporal relationship between MD and ACL injury. In our study, participants were asked to report injuries sustained within the past 12 months. In contrast, MC characteristics, specifically average MC length, were recorded at the time of questionnaire completion, without reference to the preceding year. This lack of temporal alignment between injury occurrence and menstrual status limits causal interpretation. It is therefore conceivable that some athletes had recovered from MD as a consequence of an ACL injury. Notably, athletes with ACL injuries in our sample reported higher training volumes prior to injury. Following ACL reconstruction, the subsequent reduction in training load and increased emphasis on recovery [46] may have led to improved energy availability and resumption of a regular MC. Thus, while MD was not associated with lower training volume overall, the observed association between MD recovery and ACL injury may reflect reverse causality, driven by post‐injury changes in training volume and energy balance rather than a protective effect of MD per se.

### 4.3 Knee Cartilage Injuries and PHE

The finding that in our study, female athletes undergoing regular PHE reported higher knee cartilage injury rates, may actually highlight the value of these assessments. The higher injury rates may reflect improved detection of asymptomatic or underreported joint conditions rather than a true increase in incidence. Imaging studies frequently reveal cartilage changes in asymptomatic athletes during routine PHE [47]. If left untreated both meniscal and chondral injuries can accelerate joint degeneration and lead to osteoarthritis [48]. However, shorter symptom duration, early diagnosis and treatment as well as smaller defects are associated with better outcomes and return‐to‐sport rates [49]. In our study, knee cartilage injuries were also associated with higher BMI, consistent with previous findings [48, 50], highlighting the role of modifiable risk factors in injury prevention. Preventative strategies, including structured PHE in female team sports [19, 47], may be crucial in reducing the risk of chronic joint injury and improving long‐term athlete health.

### 4.4 Strengths and Limitations

One of the main strengths of this study is the large sample size, consisting of elite female team sport athletes. Additionally, the study adhered to the IOC guidelines for reporting injuries, ensuring a standardized approach to injury reporting, which enhances consistency.

However, several limitations should be considered. First, the study relied on self‐reported injury data, which is subject to recall bias and may affect the overall validity of the findings [51]. Additionally, participants may have overlooked or forgotten mild or non‐time‐loss injuries, potentially leading to an underreporting of less severe cases. Second, there was a time‐related discrepancy between the reporting of injuries and gynaecological health: injuries were reported retrospectively for the past 12 months, whereas MC data reflected the time of questionnaire completion. This temporal mismatch limits the ability to draw causal relationships between MC characteristics and injury occurrence. Finally, MD may not have been fully captured in this study, as MD was defined solely by MC length and not assessed using more advanced diagnostic methods capable of detecting subtle forms of MD. This may have led to an underestimation of their prevalence and potential impact on injury risk.

### 4.5 Perspective

Our findings highlight the need for structured injury prevention programs, including regular PHE and professional medical support, to optimize recovery, reduce injury risks, and improve long‐term athlete health. Furthermore, they underscore the importance of interdisciplinary collaboration. However, this study also emphasizes the need for further research to better understand the role of MC characteristics, MD, and other physiological factors in injury occurrence. Future studies should investigate the effectiveness of targeted interventions such as strength training and personalized injury prevention strategies to better address the specific needs of elite female athletes across different team sports.

## 5 Conclusions

- The unexpected association between MD and lower ACL injury rates challenges existing assumptions about the impact of menstrual health on injury risk.
- The higher rates of knee cartilage injuries in female athletes undergoing regular PHE highlight their importance in early injury detection.
- Enhanced medical support structures may reduce injury risks for elite female athletes.

## Author Contributions

**Elisabeth Maria Kirschbaum:** formal analysis, funding acquisition, investigation, methodology, visualization, writing – original draft. **Jana Henke:** investigation, methodology, writing – review and editing. **Katrin Heyde:** methodology, writing – review and editing. **Kirsten Legerlotz:** methodology, supervision, writing – review and editing.

## Ethics Statement

The studies involving human participants were reviewed and approved by the ethical review board of the Institute for Applied Training Science (ER_2023.06.01_4).

## Consent

Written informed consent was obtained directly from the patient(s) involved in this study.

## Conflicts of Interest

The authors declare no conflicts of interest.

## Transparency Statement

The lead author, Elisabeth Maria Kirschbaum, affirms that this manuscript is an honest, accurate, and transparent account of the study being reported; that no important aspects of the study have been omitted; and that any discrepancies from the study as planned (and, if relevant, registered) have been explained.

## Supporting information


**Online Supplemental Material S1:** STOBE Statement Checklist.

**Online Supplemental Material S2:** Original Questions Used in German.

**Online Supplemental Material S3:** Original Questions Used in German.

**Online Supplemental Material S4:** Overview of the distribution of different hormonal profiles among female athletes in the total sample (*n* = 301). Abbeviations: HC, hormonal contraceptives; IUS, intrauterine system; OCP, oral contraceptive pill; POP, progestin only pill.

## Data Availability

The data set from the current study is available from the corresponding author on reasonable request.
