# Supplementary material for: Hormonal Contraception, Menstrual Cycle Characteristics, and Lower Limb Injuries in Elite Female Team Sports—Identifying Factors Associated With Increased Injury Prevalence: A Cross‐Sectional Study
Source: Health Sci Rep. 2026 Feb 15;9(2):e71812. doi: 10.1002/hsr2.71812 (PMC12907512; doi:10.1002/hsr2.71812)
Supplement: Supplementary file 2 — Online Supplemental Material S2: Original Questions Used in German. [file HSR2-9-e71812-s004.pdf]

Additional File 1: Original Questions Used in German

Seite 01

Allgemeine Fragen

Im folgenden Teil befragen wir Dich zu allgemeinen Angaben Deiner Person, Deiner Sportart, Deinen Trainingsumfängen und Deiner aktuellen Situation von präventiver Diagnostik.
Bitte versuche diese Fragen so genau wie möglich zu beantworten.

Die Daten werden anonym gespeichert und ein Rückschluss auf Deine Person ist nicht möglich.

AF_1 Wie alt bist Du?

- Freitext/Zahl Jahre
- Ich bin 15 Jahre alt oder jünger

Bei AF_1 = „*Ich bin 15 Jahre alt oder jünger*", beende diesen Fragebogen

AF_2 Wie groß bist Du?

- Freitext/Zahl cm

AF_3 Wie schwer bist Du?

- Freitext/Zahl kg

AF_4 Was ist Dein dominantes Bein?
Welches Bein würdest Du zum einbeinigen Abspringen benutzen?

- Links
- Rechts
- Ich habe keins
- Weiß nicht

AF_5 Welche Sportart betreibst Du?

- Basketball
- Feldhockey
- Fußball
- Handball
- Volleyball

AF_6 Hast Du aktuell einen deutschen oder ausländischen Kaderstatus?

- Nein
- Weiß nicht
- Ja, welchen?
    - Olympiakader (OK)
    - Perspektivkader (PK)
    - Teamsportkader (TK)
    - Nachwuchskader 1 (NK1)
    - Nachwuchskader 2 (NK2)
    - Landeskader (LK)
    - Ich weiß es nicht
    - Sonstigen, nämlich: Freitext

AF_7 Welches Beschäftigungsverhältnis trifft derzeit am besten auf Dich zu?
(Mehrfachauswahl möglich)

- Der Leistungssport ist mein Beruf.

- Ich bin neben dem Leistungssport erwerbstätig.
- Ich studiere neben dem Leistungssport.
- Ich mache neben dem Leistungssport eine Ausbildung.
- Ich gehe neben dem Leistungssport zur Schule.
- Andere, nämlich: Freitext

AF_8 Wie viele Stunden, die Woche betreibst Du durchschnittlich Sport?

- Freitext/Zahl Stunden Training
- Freitext/Zahl Stunden Spiel/Wettkampf (z.B.: Punktespiel, Freundschaftsspiel,...)
- Freitext/Zahl Stunden Freizeit (z.B.: Schule, Fitnessstudio,...)

AF_9 Wie viele Trainingseinheiten absolvierst Du durchschnittlich in einer Woche?

- Zahl Training (z.B.: Mannschaftstraining, Athletiktraining,...)
- Zahl Spiel/Wettkampf (z.B.: Punktspiel, Freundschaftsspiel,...)

AF_10 Wie häufig hast Du durchschnittlich die Woche Präventionstraining?
(z.B. Beinachsen-, Stabilisationstraining)

- Zahl Minuten mit (Athletik-)Trainer:in/Physiotherapeut:in
- Zahl Minuten selbstständig im Training
- Zahl Minuten selbstständig außerhalb des Trainings
- Sonstiges: Freitext
- Kein

AF_11Findet jährlich bei Dir eine Präventionsdiagnostik statt, um Kraft-, Mobilitäts- und/oder Koordinationsdefizite aufzudecken?
(z.B.: Hop/Hüpf-Test, Rumpfstütz, Isomed,...)

- Ja
- Nein
- Weiß nicht

AF_12 Findet jährlich bei Dir eine sportmedizinische Untersuchung statt?

- Ja, eine internistische Untersuchung (z.B. Blut, Herzultraschall, ...)
- Ja, eine orthopädische Untersuchung (z.B. MRT, Beweglichkeit der Gelenke, ...)
- Ja, beides
- Sonstiges: Freitext
- Nein
- Ich weiß es nicht

Seite 02

Sportartspezifisch

Im folgenden Teil befragen wir Dich detaillierter zu Deiner Sportart und Deinem aktuellen Leistungsniveau.

Bei AF_5 = „*Basketball*“ weiter mit SpS_1
Bei AF_5 = „*Feldhockey*“ oder „*Fußball*“ weiter mit SpS_2
Bei AF_5 = „*Handball*“ weiter mit SpS_3
Bei AF_5 = „*Volleyball*“ weiter mit SpS_4

SpS_1 Auf was für einer Position spielst Du die meiste Zeit?

- Point Guard
- Shooting Guard
- Small Forward
- Power Forward
- Center

→ weiter mit SpS_5

SpS_2 Auf was für einer Position spielst Du die meiste Zeit?

- Tor
- Verteidigung
- Mittelfeld
- Sturm

→ weiter mit SpS_5

SpS_3 Auf was für einer Position spielst Du die meiste Zeit?

- Tor
- Kreis
- Rückraum
- Außen

→ weiter mit SpS_5

SpS_4 Auf was für einer Position spielst Du die meiste Zeit?

- Zuspiel
- Außenangriff/Außen-Annahme
- Mittelangriff/ Mittelblocker
- Diagonal
- Libera

→ weiter mit SpS_5

SpS_5 Seit wann betreibst Du die Sportart leistungsmäßig?

- Freitext/Zahl Jahren

SpS_6 In welcher Liga spielst Du aktuell?

- Bundesliga
- 2. Bundesliga
- Andere, nämlich: Freitext

SpS_7 Seit wann spielst Du auf diesem Niveau?

- Freitext/Zahl Jahren

Page 03

Allgemein Verletzungen

Im folgenden Teil stellen wir Dir Fragen zu allgemeinen Verletzungen im Bereich der unteren Extremität (Hüfte, Knie, Fuß).

AV_1 Hattest Du innerhalb der letzten 12 Monate eine Verletzung an der unteren Extremität?

- Ja
- Nein
- Weiß nicht

Bei AV_1 = „*Nein*" oder „*Weiß nicht*", weiter mit VB_1 (Seite 04).

AV_2 Was war betroffen? [Drop-Down]

	Gelenk/Bereich *[Bitte auswählen]*	Seite: *[Bitte auswählen]*	Verletzung *[Bitte auswählen]*	Ausfallzeit *[Bitte auswählen]*
1.	• Hüfte<br>• Oberschenkel<br>• Knie<br>• Unterschenkel<br>• Sprunggelenk<br>• Fuß	• Links<br>• Rechts<br>• Beide	• Muskelverletzung<br>• Muskelprellung<br>• Kompartmentsyndrom<br>• Tendinopathie<br>• Sehnenriss<br>• Bruch (Fraktur)<br>• Ermüdungsbruch<br>• Kontusion<br>• Knorpelverletzung<br>• Kapselverletzung<br>• Kapselentzündung<br>• Schleimbeutelentzündung<br>• Kreuzbandverletzung<br>• Bänderriss<br>• Akute Instabilität<br>• Chronische Instabilität<br>• Weitere: Freitext	• 0 Tage<br>• 1-7 Tage<br>• 8-28 Tage<br>• >28 Tage
2.*	Analog 1. Verletzung	Analog 1. Verletzung	Analog 1. Verletzung	Analog 1. Verletzung
3.*	Analog 1. Verletzung	Analog 1. Verletzung	Analog 1. Verletzung	Analog 1. Verletzung
4.*	Analog 1. Verletzung	Analog 1. Verletzung	Analog 1. Verletzung	Analog 1. Verletzung

* keine Pflichtfrage

Seite 04

VB_1 Hattest Du jemals eine Verletzung des vorderen Kreuzbands?

- Ja
- Nein
- Weiß nicht

Seite 05

Angaben zur gynäkologischen Gesundheit

Fast geschafft! 😊 Verschiedene Studien zeigen, dass es einen möglichen Zusammenhang zwischen dem Menstruationszyklus und dem Auftreten von Verletzungen des vorderen Kreuzbands gibt. Auch wenn Du noch keine Verletzung hattest, helfen Deine Angaben spätere Präventionsstudien besser zu planen. Deshalb stellen wir Dir auf den folgenden Seiten einige medizinische Fragen zu Deinem Menstruationszyklus und Beckenboden. Darüber hinaus erheben wir Daten über Deine Nutzung hormoneller Verhütungsmittel.
Für unsere Studie ist von entscheidender Bedeutung, dass Du die kommenden Fragen so genau und ehrlich wie möglich beantwortest.

BB_1 Wie häufig verlierst Du ungewollt Harn?

- Niemals
- Ungefähr 1 mal pro Woche oder weniger
- 2 oder 3 mal pro Woche
- Ungefähr einmal am Tag
- Mehrmals am Tag
- Ständig

BB_2 Wir würden gerne wissen, wieviel Harn Du deiner Meinung nach ungewollt verlierst?
(Unabhängig davon, ob Du Vor-/Einlagen trägst oder nicht)

- Kein Harnverlust
- Eine kleine Menge Harn
- Eine mittlere Menge Harn
- Eine große Menge Harn

BB_3 Wie sehr beeinträchtigt generell der ungewollte Harnverlust Deinen Alltag?
Bitte markiere eine Zahl zwischen 1 (überhaupt nicht) und 10 (ein schwerwiegendes Problem)

○ 1 ○ 2 ○ 3 ○ 4 ○ 5 ○ 6 ○ 7 ○ 8 ○ 9 ○ 10

BB_4 Wann verlierst Du ungewollt Harn?
(Mehrfachauswahl möglich)

- Niemals – kein ungewollter Harnverlust
- Harnverlust vor Erreichen der Toilette
- Harnverlust beim Husten oder Nießen
- Harnverlust während des Schlafens
- Harnverlust bei körperlicher Betätigung/sportlicher Aktivität
- Harnverlust nach dem Wasserlassen und Wiederankleiden
- Harnverlust ohne erkennbare Ursache
- Ständiger ungewollter Harnverlust

<u>Seite 06</u>

AG_1 Wie oft wirst Du von einer Frauenärztin/ einem Frauenarzt untersucht (ohne Schwangerschaft)?
- Mehr als 2x im Jahr
- 2x im Jahr
- 1x im Jahr
- Alle 2 Jahre
- Seltener als alle 2 Jahre
- Nie

AG_2 Hast Du gynäkologische Vorerkrankungen (Eierstockzyste, Polyzystisches Ovar Syndrom (PCOS), Menstruationsstörungen, starke Menstruationsschmerzen, …)?
- Ja
  - Wenn ja, welche? Freitext
- Nein

AM_1 Hattest Du bereits deine erste Regelblutung (Menarche)?
- Ja
- Nein

Bei AM_1 = „*Nein*", beende diesen Fragebogen

AM_2 Wie alt warst Du, als Du Deine Regelblutung (Menarche) zum ersten Mal hattest?
- Freitext/Zahl Jahre

AM_3 Mit welchem Abstand tritt gewöhnlich Deine Regelblutung auf (jeweils 1. Tag der Blutung)?
- Freitext/Zahl Tage
- Aktuell nicht zutreffend, weil: Freitext

AM_4 Wie viele Tage dauert durchschnittlich Deine Regelblutung?
- Freitext/Zahl Tage

AM_5 Wie häufig musst Du während der Regelblutung Ihre Hygieneartikel (Binde, Tampon, Menstruationstasse, …) wechseln?
- Alle 5 bis 6 Stunden
- Alle 3 bis 4 Stunden
- Alle 1 bis 2 Stunden
- Sonstiges: Freitext

AM_6 Wie oft hast Du Schmerzen oder Beschwerden während der Regelblutung?
- Bei jeder Regelblutung
- Bei jeder zweiten Regelblutung
- Bei jeder dritten Regelblutung
- Nur in seltenen Ausnahmefällen
- Nie

AM_7 Wann hast Du Schmerzen oder Beschwerden während der Regelblutung?
- Nur am ersten Tag
- 2 Tage
- Über die gesamte Blutungsdauer
- Nie

AM_8 Wie oft nimmst Du während deiner Regelblutung Schmerzmittel (z.B. Ibuprofen, Paracetamol, Buscopan oder ähnliches) ein?

- Bei jeder Regelblutung
- Bei jeder zweiten Regelblutung
- Bei jeder dritten Regelblutung
- Nur in seltenen Ausnahmefällen
- Nie

AM_9 Hast du Schmerzen oder Beschwerden 1-4 Tage vor der Regelblutung?

- Ja
- Nein
- Weiß nicht

AM_10 Ist es bei Dir schon einmal vorgekommen, dass deine Regelblutungen einen Abstand von mehr als 5 Wochen hatten?

- Ja, aktuell
- Ja, in der Vergangenheit
- Nein

Bei AM_10 = „*Ja, aktuell*“ oder „*Ja, in der Vergangenheit*“ weiter mit AM_11
Bei AM_10 = „*Nein*“ weiter mit AM_14

AM_11 Ist es bei Dir schon einmal vorgekommen, dass Du mindestens 3 Monate in Folge keine Regelblutung hattest (Schwangerschaft ausgeschlossen)?

- Ja, aktuell
- Ja, in der Vergangenheit
- Nein

AM_12 Wann war Deine Regelblutung unregelmäßig (Abstand mehr als 5 Wochen) oder hat ausgesetzt (min. 3 Monate)? *(Mehrfachauswahl möglich)*

- Während der Wettkampfphase.
- Im Trainingsbetrieb außerhalb der Wettkampfphase.
- Während der trainingsfreien Zeit.
- Ich weiß nicht mehr, in welcher Phase.
- Sonstiges: Freitext

AM_13 Hattest Du zusätzliche Probleme als Deine Regelblutung unregelmäßig war oder länger als 3 Monate ausgesetzt hat (anfälliger für Verletzungen, häufiger krank, ...)?

- Ja
  - Welche? Freitext
- Nein

AM_14 Nutzt Du aktuell ein hormonelles Verhütungsmittel?

- Nein
- Ja
  - Welches? Bitte gib auch den Namen des Präparates an.
    - Verhütungspille: Name/Freitext
    - Minipille: Name/Freitext
    - Hormonspirale: Name/Freitext

        - Kupferspirale: Name/Freitext
        - Verhütungsring: Name/Freitext
        - Hormonstäbchen/-pflaster: Name/Freitext
        - Sonstiges: Freitext

Bei AM_14 = „*Nein*“ weiter mit AM_17

AM_15 Aus welchen Gründen nutzt Du hormonelle Verhütungsmittel?
(z.B. Verhütung, bessere Planbarkeit der Regelblutung, Behandlung von anderen Beschwerden, …)

- Freitext

AM_16 Hast Du Beschwerden unter der hormonellen Verhütung?

- Ja
    - Welche? Freitext
- Nein

AM_17 Dokumentierst Du Deinen Menstruationszyklus (z.B. Zyklus-App, Zyklustagebuch,…)?

- Ja
    - Wie? Freitext
- Nein
