# Supplementary material for: Hormonal Contraception, Menstrual Cycle Characteristics, and Lower Limb Injuries in Elite Female Team Sports—Identifying Factors Associated With Increased Injury Prevalence: A Cross‐Sectional Study
Source: Health Sci Rep. 2026 Feb 15;9(2):e71812. doi: 10.1002/hsr2.71812 (PMC12907512; doi:10.1002/hsr2.71812)
Supplement: Supplementary file 3 — Online Supplemental Material S3: Original Questions Used in German. [file HSR2-9-e71812-s002.pdf]

Additional File 1: Original Questions Used Translated in English

Page 01

General Questions

In the following section, we will ask you about general information about yourself, your sport, your training volumes, and your current situation regarding injury risk factor assessments.
Please try to answer these questions as accurately as possible.

The data will be stored anonymously, and no conclusions can be drawn about your identity.

AF_1 How old are you?

- Open-ended/integer years
- I am 15 years old or younger

If AF_1 = „*I am 15 years old or younger*“, end this questionnaire

AF_2 How tall are you?

- Open-ended/integer cm

AF_3 How much do you weigh?

- Open-ended/integer kg

AF_4 What is your dominant leg?
Which leg would you use for a single-leg jump take off?

- Left
- Right
- I don't have one
- Don't know

AF_5 Which sport do you play?

- Basketball
- Field hockey
- Football
- Handball
- Volleyball

AF_6 Are you currently a German or international squad member?

- No
- Don't know
- Yes, which squad status?
    - Olympiakader (OK)
    - Perspektivkader (PK)
    - Teamsportkader (TK)
    - Nachwuchskader 1 (NK1)
    - Nachwuchskader 2 (NK2)
    - Landeskader (LK)
    - Don't know
    - Other, namely: open-ended

AF_7 Which employment situation currently applies best to you?
(Multiple answers possible)

- I am a professional athlete.

- I am employed alongside sports.
- I am studying alongside sports.
- I am doing an apprenticeship alongside sports.
- I am going to school alongside sports.
- Other, namely: open-ended

AF_8 How many hours do you train on average per week?
- Open-ended/integer h of training
- Open-ended/integer h of game/competition (e.g. match, friendly match,...)
- Open-ended/integer h of leisure activity (e.g. PE, fitness studio,...)

AF_9 How many training sessions do you complete on average per week?
- Open-ended/integer training (e.g. team training, strength & conditioning,...)
- Open-ended/integer game/competition (e.g. match, friendly match,...)

AF_10 How often do you do preventive training per week?
(e.g. leg-axis training, stabilisation training)
- Open-ended/integer minutes with (S&C) coach/physiotherapist
- Open-ended/integer minutes independently during training
- Open-ended/integer minutes independently outside of training
- Other: Open-ended
- None

AF_11 Do you undergo annual injury risk factor assessment to detect strength, mobility, and/or coordination deficits?
(e.g..: hop/jump test, plank, Isomed,...)
- Yes
- No
- Don't know

AF_12 Do you undergo an annual sports medical examination?
- Yes, an internal examination (e.g. blood status, cardiac ultrasound, ...)
- Yes, an orthopaedic examination (e.g. MRI, Joint mobility, ...)
- Yes, both
- Other: Open-ended
- None
- Don't know

Page 02

Sport-specific questions
In the following section, we will ask you more detailed questions about your sport and your current performance level.

If AF_5 = „*Basketball*" continue with SpS_1
If AF_5 = „*Field hockey*" or „*Football*" continue with SpS_2
If AF_5 = „*Handball*" continue with SpS_3
If AF_5 = „*Volleyball*" continue with SpS_4

SpS_1 Which position do you play most of the time?
- Point Guard
- Shooting Guard
- Small Forward
- Power Forward
- Centre

→ continue with SpS_5

SpS_2 Which position do you play most of the time?
- Goalkeeper
- Defence
- Midfield
- Forward

→ continue with SpS_5

SpS_3 Which position do you play most of the time?
- Goalkeeper
- Pivot
- Backcourt
- Wing

→ continue with SpS_5

SpS_4 Which position do you play most of the time?
- Setter
- Outside hitter
- Middle blocker/hitter
- Diagonal
- Libero

→ continue with SpS_5

SpS_5 Since when have you been playing this sport at a high-performance level?
- Open-ended/integer years

SpS_6 In which division do you currently play?
- 1st Bundesliga
- 2nd Bundesliga
- Other, namely: Open-ended

SpS_7 Since when have you been playing at this level?
- Open-ended/integer years

General Injuries

In the following section, we will ask you questions about general injuries in the lower extremities (hip, knee, foot).

AV_1 Have you had an injury to the lower extremity **within the last 12 months**?

- Yes
- No
- Don't know

If AV_1 = „*No*" or „*Don't know*", continue with VB_1 (page 04).

AV_2 What was affected? [Drop-Down]

	Body area *[Please select]*	Side: *[Please select]*	Injury *[Please select]*	Time loss *[Please select]*
1.	• Hip<br>• Upper thigh<br>• Knee<br>• Lower thigh<br>• Ankle<br>• Foot	• Left<br>• Right<br>• Both	• Muscle injury<br>• Muscle contusion<br>• Muscle compartment syndrome<br>• Tendinopathy<br>• Tendon rupture<br>• Fracture<br>• Bone stress injury<br>• Bone contusion<br>• Cartilage injury<br>• Capsular injury<br>• Synovitis/Capsulitis<br>• Bursitis<br>• ACL injury<br>• Joint sprain<br>• Acute instability<br>• Chronic instability<br>• Other: open-ended	• 0 days<br>• 1-7 days<br>• 8-28 days<br>• >28 days
2.*	Same as 1st injury	Same as 1st injury	Same as 1st injury	Same as 1st injury
3.*	Same as 1st injury	Same as 1st injury	Same as 1st injury	Same as 1st injury
4.*	Same as 1st injury	Same as 1st injury	Same as 1st injury	Same as 1st injury

* optional

Page 04

VB_1 Have you ever had an anterior cruciate ligament (ACL) injury?

- Yes
- No
- Don’t know

Page 05

Gynaecological Health

Almost done! 😊 Various studies show that there is a possible connection between the menstrual cycle and the occurrence of ACL injuries.
Even if you have not had an injury yet, your information helps to better plan future prevention studies. Therefore, on the following pages, we will ask you some medical questions about your menstrual cycle and pelvic floor. In addition, we will collect data about your use of hormonal contraceptives.
For our study, it is crucial that you answer the upcoming questions as accurately and honestly as possible.

BB_1 How often do you leak urine?

- Never
- About once a week or less often
- Two or three times a week
- About once a day
- Several times a day
- All the time

BB_2 We would like to know how much urine do you think leaks. How much urine do you usually leak (whether you war protection or not)?

- None
- A small amount
- A moderate amount
- A large amount

BB_3 Overall, how much does leaking urine interfere with your everyday life?
Please mark a number between 1 (not al all) an 10 (a great deal)

○ 1 ○ 2 ○ 3 ○ 4 ○ 5 ○ 6 ○ 7 ○ 8 ○ 9 ○ 10

BB_4 When does urine leak?
(Please tick all that apply to you)

- Never – urine does not leak
- Leaks before you can get the toilet
- Leaks when you cough or sneeze
- Leaks when you are asleep
- Leaks when you are physically active/exercising
- Leaks when you have finished urinating and are dressed
- Leaks for no obvious reason
- Leaks all the time

<u>Page 06</u>

AG_1 How often do you have gynecological appointments (without pregnancy)?

- More than 2x a year
- 2x a year
- 1x a year
- Every 2 years
- Less than every 2 years
- Never

AG_2 Do you have any previous gynecological diseases (ovarian cyst, polycystic ovary syndrome (PCOS), menstrual disorders, severe menstrual pain, …)?

- Yes
    - If yes, which ones? Open-ended
- No

AM_1 Have you already had your first menstrual period (menarche)?

- Yes
- No

If AM_1 = „*No*", end this questionnaire

AM_2 How old were you when you had your first menstrual period (menarche)?

- Open-ended/Integer years

AM_3 What is the usual amount of days between the first day of period and the first day of your next's period?

- Open-ended/interger days
- Currently no applicable, because: Open-ended

AM_4 How many days lasts you period normally?

- Open-ended/interger days

AM_5 How often do you change the sanitary products (pad, tampon, menstrual cup, …) during your period?

- Every 5 to 6 hours
- Every 3 to 4 hours
- Every 1 to 2 hours
- Other: Open-ended

AM_6 How often do you have pain or discomfort during your period?

- Every period
- Every second period
- Every third period
- Only in rare exceptional cases
- Never

AM_7 When do you have period pain? When does the pain occur?

- First day only
- 2 days
- Over the entire bleeding period
- Never

AM_8 How often do you use painkillers (e.g. ibuprofen, paracetamol, buscopan or similar) to manage pain during your period?

- Every period
- Every second period
- Every third period
- Only in rare exceptional cases
- Never

AM_9 Do you have pain or discomfort 1-4 days prior your period?

- No
- Yes
- Don't know

AM_10 Have you ever not had a period for more than 5 weeks?

- Yes, currently
- Yes, in the past
- No

If AM_10 = „*Yes, currently*" or "*Yes, in the past*" continue with AM_11
If AM_10 = "*No*" continue with AM_14

AM_11 Have you ever not had a period for more than 3 months?

- Yes, currently
- Yes, in the past
- No

AM_12 When was your period irregular (more than 5 weeks apart) or stopped (at least 3 months)? (Multiple selection possible)

- During the competition period
- During training outside the competition period.
- During the non-competitive training period.
- I don't remember in which period.
- Other: Open-ended

AM_13 Did you have additional problems when your periods were irregular or stopped for more than 3 months (more sensitive to injuries, more often ill, …, …)?

- Yes
  - Which one? Open-ended
- No

AM_14 Do you currently use any hormonal contraceptives?

- No
- Yes
  - Which one? Please also specify the name of the preparation.
    - Contraceptive pill: Name/Open-ended
    - Minipill: Name/Open-ended
    - Hormone IUD: Name/ Open-ended
    - Copper IUD: Name/ Open-ended
    - Contraceptive ring: Name/Open-ended

        - Hormone sticks/patches: Name/Open-ended
        - Other, namely: Open-ended

If AM_14 = "*No*" continue with AM_17

AM_15 Why do you use hormonal contraceptives (Contraception, better period prediction, treatment of other complaints, ...)

- Open-ended

AM_16 Do you have any symptoms under hormonal contraception?

- Yes
    - Which one? Open-ended
- No

AM_17 Do you currently document/track your menstrual cycle (e.g. cycle app, cycle diary,...)?

- Yes
    - How? Open-ended
- No
