# Supplementary figures and images for: Hormonal Contraception, Menstrual Cycle Characteristics, and Lower Limb Injuries in Elite Female Team Sports—Identifying Factors Associated With Increased Injury Prevalence: A Cross‐Sectional Study
Source: Health Sci Rep. 2026 Feb 15;9(2):e71812. doi: 10.1002/hsr2.71812 (PMC12907512; doi:10.1002/hsr2.71812)

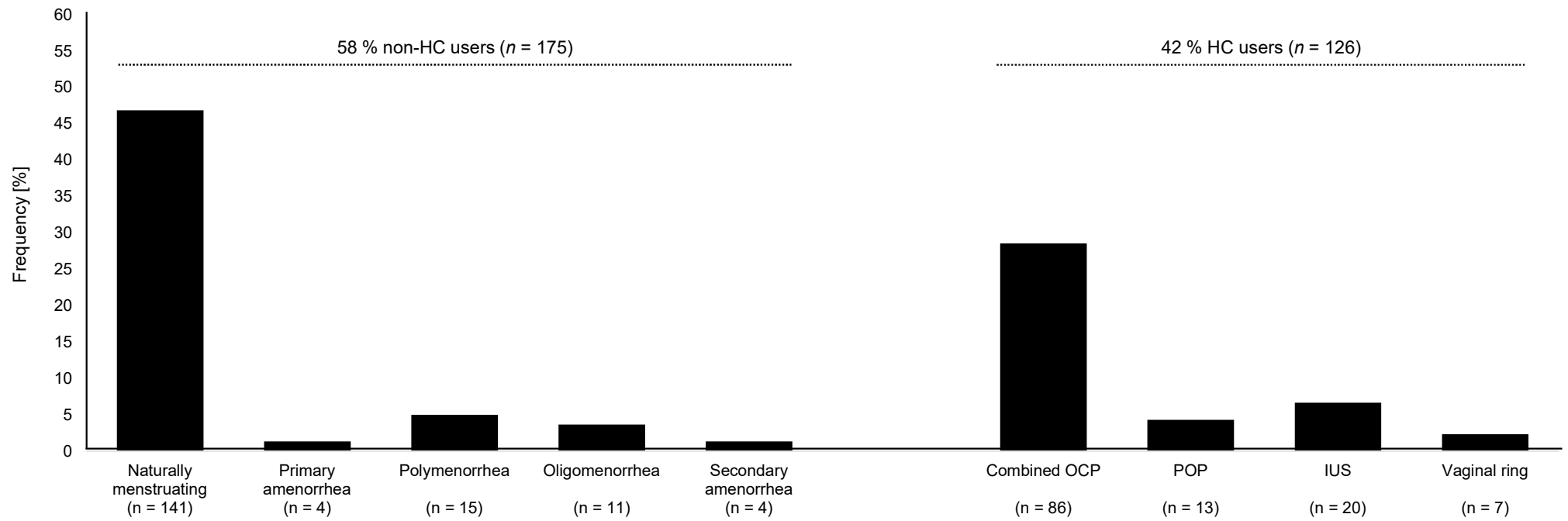

Supplement: Supplementary file 4 — Online Supplemental Material S4: Overview of the distribution of different hormonal profiles among female athletes in the total sample (n = 301). Abbeviations: HC, hormonal contraceptives; IUS, intrauterine system; OCP, oral contraceptive pill; POP, progestin only pill. [file HSR2-9-e71812-s001.pdf]
